# Increasing Voter Participation Through Health Care–Based Voter Registration

**DOI:** 10.1001/jamahealthforum.2024.1563

**Published:** 2024-06-21

**Authors:** Katherine McCabe, Yinlu Zhu, Simar S. Bajaj, Alister F. Martin

**Affiliations:** 1 Department of Political Science, Rutgers University, New Brunswick, New Jersey; 2 Vot-ER, Boston, Massachusetts; 3 Harvard University, Cambridge, Massachusetts; 4 Department of Emergency Medicine, Massachusetts General Hospital, Boston

## Abstract

**Importance:**

Young people and historically marginalized racial and ethnic groups are poorly represented in the democratic process. Addressing voting inequities can make policy more responsive to the needs of these communities.

**Objective:**

To assess whether leveraging health care settings as venues for voter registration and mobilization is useful, particularly for historically underrepresented populations in elections.

**Design, Setting, and Participants:**

In 2020, nonpartisan nonprofit Vot-ER partnered with health care professionals and institutions to register people to vote. This cross-sectional study analyzed the demographics and voting behavior of people mobilized to register to vote in health care settings, including hospitals, community health centers, and medical schools across the US. The age and racial and ethnic identity data of individuals engaged through Vot-ER were compared to 2 national surveys of US adults, including the 2020 Cooperative Election Study (CES) and the 2020 American National Election Study (ANES).

**Exposure:**

Health care–based voter registration.

**Main Outcomes and Measures:**

The main outcomes were age composition, racial and ethnic composition, and voting history.

**Results:**

Of the 12 441 voters contacted in health care settings, 41.9% were aged 18 to 29 years, 15.9% were identified as African American, 9.6% as Asian, 12.7% as Hispanic, and 60.4% as White. This distribution was significantly more diverse than the racial and ethnic distribution of the ANES (N = 5447) and CES (N = 39 014) samples, of which 72.5% and 71.19% self-identified as White, respectively. Voter turnout among health care–based contacts increased from 61.0% in 2016 to 79.8% in 2020, a turnout gain (18.8–percentage point gain) that was 7.7 percentage points higher than that of the ANES sample (11.1–percentage point gain). Demographically, the age distribution of voters contacted in health care settings was significantly different from the ANES and CES samples, with approximately double the proportion of young voters aged 18 to 29 years.

**Conclusion and Relevance:**

This cross-sectional study suggests that health care–based voter mobilization reaches a distinctly younger and more racially and ethnically diverse population relative to those who reported contact from political campaigns. This analysis of the largest health care–based voter mobilization effort points to the unique impact that medical professionals may have on voter registration and turnout in the 2024 US elections. In the long term, health equity initiatives should prioritize expanding voting access to address the upstream determinants of health in historically marginalized communities.

## Key Points

**Question:**

Can health care–based voter mobilization efforts help reach populations underrepresented in US elections?

**Findings:**

This cross-sectional study compared data from individuals reached in health care settings before the 2020 US elections with 2 national surveys of US adults, including the 2020 Cooperative Election Study and the 2020 American National Election Study. Of the 12 441 health care–based contacts, a significantly larger proportion were young (41.9%) and racial and ethnic minority individuals (39.6%) relative to the nationally representative data.

**Meaning:**

Health care settings may register and mobilize younger and more racially and ethnically diverse voters, suggesting medical professionals can play an important role in voter registration and turnout during the 2024 US elections.

## Teaser

This cross-sectional study assesses whether leveraging health care settings as venues for voter registration and mobilization is useful, particularly for historically underrepresented populations in elections.

## Introduction

In the US, 51 million voters are not registered to vote, nearly a quarter of those eligible. Unregistered voters are disproportionately represented by racial and ethnic minority and lower-income individuals.^1^ Debates on equity and social determinants of health are undermined without inclusion of the broader citizenry. Indeed, Rodriguez and colleagues^2^ found that almost 20 state-level elections from 1970 to 2004 may have had a different outcome if Black people of voting age had the same mortality profile as their White counterparts.

Previous studies have suggested that many eligible voters lack the time or understanding to navigate the registration process but are receptive to being invited to register to vote. Given that emergency departments disproportionately care for low-income, minority, and uninsured individuals and that these populations are also less likely to vote,^3^ the 501(c)3 nonprofit Vot-ER was founded in 2019 to support patients’ voter registration and electoral participation in health care settings.

Since then, Vot-ER’s reach has expanded beyond the emergency department, with academic medical centers, medical schools, and federally qualified health centers using Vot-ER’s posters and flyers to provide passive, optional opportunities for voter registration. Vot-ER also equips health care professionals nationwide with free voter registration badge backers, which have a QR code directing patients to check their registration, register to vote, or request an absentee ballot. Between July and October 2020, more than 24 000 health care professionals ordered voter registration badges. By Election Day 2022, Vot-ER tools were used more than 75 000 times.

In this cross-sectional study, we compared voting history and age, racial, and ethnic distributions of US adults contacted through Vot-ER’s health care–based outreach to adults contacted by traditional political campaigns. We sought to determine how mobilizing people to vote in health care settings could recover inequalities in opportunity, reaching populations that are lower-propensity voters or otherwise underrepresented in election mobilization efforts.

## Methods

This cross-sectional study analyzed 3 databases from 2020, including Vot-ER’s Registration Tool Contacts, the Cooperative Election Study (CES), and the American National Election Study (ANES). The Rutgers University Institutional Review Board provided a nonhuman participant research determination, so this secondary analysis of the Vot-ER and US national survey data was exempt from review and informed consent. We followed the Strengthening the Reporting of Observational Studies in Epidemiology (STROBE) reporting guideline.

### 2020 Vot-ER Registration Tool Contacts

This data set includes 14 564 individuals who provided contact information through one of Vot-ER’s voter registration tools, checking their existing registration status or registering to vote for the first time, before the US elections in November 2020. The analysis sample includes only Vot-ER contacts who were able to be matched to a record in a national file of voters and voting-age individuals assembled by an organization that uses public voting records and consumer databases to enable studies of political participation and vote choice (TargetSmart). This matched sample of contacts includes each individual’s age, racial and ethnic identity, voting record in 2020, and voting history from 2016. The main analysis limits the sample to those who were 18 years or older by Election Day in 2020 and thus age-eligible to vote, but we further restricted the sample to those age-eligible to vote in 2016 when examining voting history.

### 2020 Cooperative Election Study

The 2020 CES is a national 2-wave panel survey of US adults before and after the 2020 general election. The preelection wave was in the field from September 29, 2020, to November 2, 2020. The postelection wave was in the field from November 8, 2020, to December 14, 2020. The CES asked a range of questions related to political participation and attitudes, alongside standard demographic questions including age and racial and ethnic identity. The CES validated the respondents’ 2020 voter turnout, identifying whether a respondent had a verifiable record of voter registration or voting in the election using a third-party vendor (Catalist National Database). For comparability with the Vot-ER data, the analysis focused on those CES respondents who were matched to the Catalist voter file. To identify a comparable CES sample of individuals contacted about voter registration and turnout, we conducted a subanalysis, including only CES respondents who self-reported contact by a political campaign or candidate.

### 2020 American National Election Study

The 2020 ANES is a 2-wave panel survey of US adults fielded before and following the 2020 general election. The preelection wave was administered between August 18, 2020, and November 3, 2020. The postelection wave was conducted between November 9, 2020, and January 4, 2021. The ANES included voter turnout information validated by third-party vendors for both the 2016 and 2020 elections, along with respondents’ self-reported age and racial and ethnic identity. From the ANES survey data, we included only respondents who were matched to records in public voter files.

To identify a comparable ANES sample of individuals contacted about voter registration and turnout, we conducted subanalyses, including only respondents who self-reported being contacted by (1) a campaign or some other group for persuasion about voting for a particular candidate and (2) by someone about registering to vote or getting out to vote. Finally, the ANES included 2016 election voting data, offering the ability to compare whether the population reached by Vot-ER tended to have a lower or higher baseline propensity to vote in the 2020 elections, as compared with potential voters more broadly.

## Results

**Figure 1.**
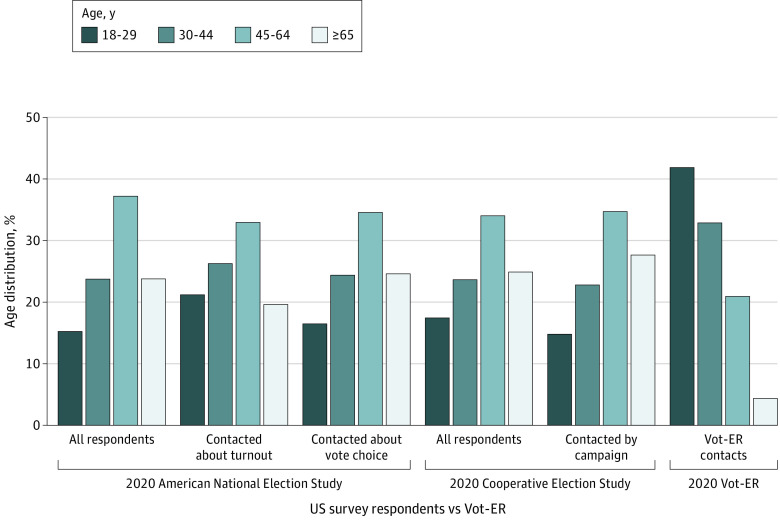
Age Distribution Between Vot-ER and Political Campaign Contacts in 2020 This bar graph shows the age percentage distribution of Vot-ER contacts reached in a health care setting compared to respondents in 2 US national election surveys: the 2020 Cooperative Election Study and the 2020 American National Election Study.

A total of 12 441 individuals from the Vot-ER Registration Tool Contacts met the inclusion criteria, as well as 39 014 individuals from the 2020 CES and 5447 individuals from the 2020 ANES. Young people aged between 18 and 29 years made up a larger share of Vot-ER contacts (41.9%) compared to the CES (17.4%) and ANES (15.2%) samples (Figure 1). In a subanalysis of the CES respondents who self-reported being contacted by a political campaign or candidate (n = 26 840), young people made up 14.8% of the sample. Similarly, in subanalyses of the ANES, focusing on respondents who self-reported being contacted about voting for a particular candidate (n = 2371) or being contacted about registering to vote or getting out to vote (n = 2638), young people made up 16.4% and 21.2% of the ANES samples, respectively, which was approximately half the proportion of young people in the Vot-ER sample.

### Race and Ethnicity

**Figure 2.**
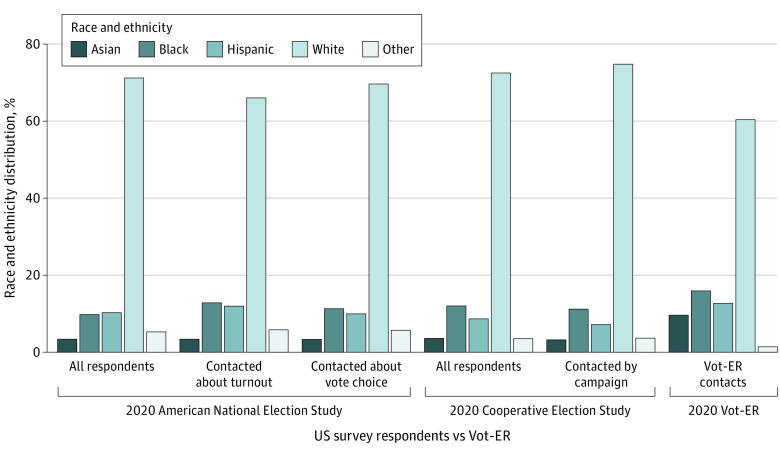
Race and Ethnicity Distribution Between Vot-ER and Political Campaign Contacts in 2020 This bar graph shows the race and ethnicity percentage distribution of Vot-ER contacts reached in a health care setting compared to respondents in 2 US national election surveys: the 2020 Cooperative Election Study (CES) and the 2020 American National Election Study (ANES). Race and ethnicity data for Vot-ER data were derived from TargetSmart, an organization that uses public voting records and consumer databases to enable research, whereas race and ethnicity data in CES and ANES were self-reported. For Vot-ER data, the other category included multiracial, Native American, “other” (as reported in the database), or uncoded. For CES, the group other includes Native American, 2 or more races, Middle Eastern, or self-reported “other.” For the ANES, other includes Native American/Alaska Native or self-reported other race and multiracial.

A smaller share of the Vot-ER contacts in 2020 was White (60.4%), compared to 72.5% of CES and 71.19% of ANES respondents who self-identified as White (Figure 2). Vot-ER contacts had an especially high Asian composition, with 9.6% of contacts identified as Asian, which is 3.0 times that of the CES sample and 2.8 times that of the ANES sample. In all subanalyses of the CES and ANES of survey respondents who reported being contacted by a campaign about vote choice or turnout, Asian representation was less than 3.4%.

Vot-ER contacts also tended to have comparable or slightly higher representation of African American and Hispanic individuals. Namely, 15.9% of Vot-ER contacts were identified as African American in the database, compared to 9.8% of the ANES sample and 12.0% of the CES sample who self-reported as Black. Additionally, 12.7% of Vot-ER contacts were identified as Hispanic in the database, which is slightly higher than representation among all ANES respondents (10.2%) and those contacted by campaigns about vote choice (10.0%), although comparable to those contacted about turnout (12.0%). The Vot-ER share is also slightly higher than representation among all CES respondents (8.4%) and those who reported being contacted by a campaign (7.2%).

### Voter Turnout

Examining voter turnout history, people reached through Vot-ER during the 2020 election campaign had a significantly lower turnout propensity based on 2016 voting records compared to the ANES national sample of US adults. Among Vot-ER contacts who were 18 years or older in 2016 (n = 11 632), turnout was 61.0% in the 2016 election, compared to 74.7% among the sample of 2020 ANES respondents in 2016 (n = 5192) or a smaller subsample of those who were contacted about voting in 2020 (73.5%). By extension, Vot-ER contacts saw large turnout gains in 2020, with proportions increasing to 79.8% in 2020, while turnout among ANES respondents increased to 85.8%. In other words, Vot-ER contacts saw an increase in turnout rate over time that was 7.7 percentage points larger than that of the ANES sample. These increases in voting among Vot-ER contacts occurred across racial and ethnic groups. Among those age-eligible in 2016, turnout rates in 2016 among Vot-ER contacts were 58.5% for African American contacts, 53.2% for Asian contacts, 50.4% for Hispanic contacts, and 65.2% for White contacts. The 2020 turnout rates for these racial groups were 72.1%, 83.4%, 83.0%, and 71.7%, respectively.

## Discussion

Underserved populations, historically, are left out of voter registration drives by government offices, state election websites, and political campaigns. For example, leading up to the 2020 election, Asian and Latino eligible voters, as well as people with lower levels of political activism more generally, were less likely to report campaign outreach compared to other eligible voters.^4,5^ In this cross-sectional study, we demonstrated that health care–based voter engagement can reach a significantly younger and more racially and ethnically diverse population than the general voting-age population and those contacted by political campaigns.

Several explanations for the unique reach of health care–based voter mobilization are possible. First, pediatricians and pediatric institutions comprised 13% to 17% of health care professionals and organizations that implemented Vot-ER’s voter registration resources in 2020.^6^ Their participation was bolstered by the American Academy of Pediatrics, which hosted an extensive Get Out the Vote initiative in 2020 and may have contributed to the significantly greater mobilization of younger individuals among the Vot-ER cohort. Health care–based civic engagement may have also mobilized a younger subset of the health care workforce, with 80 medical schools participating in a nationwide voter registration competition.^7^ Implementation of Vot-ER tools varied across different sites, with some using individual Healthy Democracy Kits (HDKs) consisting primarily of the badge backer, and others using institutional HDKs that included flyers and posters. Individual HDKs were predominantly used by medical students and physicians from academic institutions, while institutional HDKs were primarily used by nonacademic institutions, pediatric-only institutions, and federally qualified health centers.^6^

The greater racial and ethnic diversity among Vot-ER contacts may be associated with health care institutions participating in voter registration efforts in areas of the country with a higher-than-average percentage of racial and ethnic minority residents.^6^ The annual rates for visiting the emergency department are almost twice as high for African American or Black patients than for White patients in the US.^8^ More broadly, nonurgent visits to hospital emergency departments are more likely among non-Hispanic Black and younger individuals with Medicaid or no insurance.^9^ This overlaps with populations more likely to face barriers to voting, namely racial and ethnic minority groups, lower-income individuals, and young people.^10^

Although we did not have data on the health status of people in the Vot-ER, ANES, or CES databases, participants mobilized in health care settings would most likely have poorer health than the general US adult population. Indeed, previous data show that patients with disabilities, depression, heart disease, and other chronic conditions are less likely to vote than their counterparts.^11,12,13^ With these communities’ electoral participation hampered by poor health, a self-perpetuating cycle persists in which policy is increasingly unable to serve people who need high-quality, low-cost health care the most. Health care–based voter registration is one potential response to this misalignment, more successfully engaging lower-propensity voters than traditional political campaigns.

### Limitations

First, Vot-ER data relied on TargetSmart’s reports of an individual’s racial and ethnic identity, while CES and ANES survey data relied on self-reported race and ethnicity. Due to failed matches to TargetSmart’s database, approximately 12% of Vot-ER contacts were thus excluded. The current study likely underestimated the actual percentage of young and lower-income people reached in health care settings given these populations’ disproportionate exclusion by TargetSmart’s unregistered voting age records. The data also likely underestimated the total number of individuals contacted via health care outreach because voters who chose to register to vote through their state’s election page rather than through Vot-ER tools would not have been included in this analysis.

Finally, a causal relationship between health care–based outreach and individuals registering to vote or voting in the 2020 election cannot be determined because contact was not randomly assigned. We cannot observe what would have happened if the contact had not occurred. However, lower 2016 turnout among contacts in the Vot-ER database suggests that people who benefited from health care–based voter registration were lower-propensity voters relative to the US national survey samples. Voters contacted in health care settings ultimately demonstrated a significantly higher increase in turnout rates in 2020 than the standard US population.

## Conclusions

This cross-sectional study suggests that health care settings may serve as effective venues for voter registration and mobilization programs because these settings routinely engage subsets of the population historically excluded from the democratic process. These settings also hold a unique power to encourage civic engagement as health care professionals may be viewed as trusted messengers. In 2023, 325 voting restriction bills were introduced in 45 states with 17 already enacted into law.^14^ These restrictive voting laws disproportionately impact younger and racial and ethnic minority individuals with poorer health outcomes, likely reducing the turnout of these individuals during the 2024 US election.^15,16^

In the long term, initiatives addressing health inequities should prioritize expanding voting access to support the long-term empowerment of marginalized communities. In the short term, however, this study demonstrates how physicians can play an important role in ensuring the political inclusion of marginalized groups by helping patients register to vote.

Even without claiming that health care–based mobilization can fully turn a nonvoter into a voter, the form of voter education provided through this mobilization can nonetheless reduce barriers to voting by lowering the costs of accessing information about elections and motivating people to vote by signaling that someone in their community cares about their opinion.^15^ Future research could randomly assign health care–based outreach to different patient populations or communities to help identify the causal effect of this outreach on voting.

## Notes

Supplement.
Data Sharing Statement
