# Supplementary material for: Increasing Voter Participation Through Health Care–Based Voter Registration
Source: JAMA Health Forum. 2024 Jun 21;5(6):e241563. doi: 10.1001/jamahealthforum.2024.1563 (PMC11193121; doi:10.1001/jamahealthforum.2024.1563)
Supplement: Supplement. — Data Sharing Statement [file jamahealthforum-e241563-s001.pdf]

Data Sharing Statement

McCabe. Increasing Voter Participation Through Health Care–Based Voter Registration. *JAMA Health Forum*. Published June 21, 2024. doi:10.1001/jamahealthforum.2024.1563

Data
Data available: No

Additional Information
Explanation for why data not available: Data from the ANES are available here: https://electionstudies.org/. Data from the CES are available here: https://cces.gov.harvard.edu/data. Voting record data from the healthcare mobilization will not be shared.
